# Association of the interleukin 1 beta gene and brain spontaneous activity in amnestic mild cognitive impairment

**DOI:** 10.1186/1742-2094-9-263

**Published:** 2012-12-01

**Authors:** Liying Zhuang, Xiaoyan Liu, Xiaohui Xu, Chunxian Yue, Hao Shu, Feng Bai, Hui Yu, Yongmei Shi, Zhijun Zhang

**Affiliations:** 1 Medical School of Southeast University, 87 Dingjiaqiao Road, Nanjing, Jiangsu, 210009, China; 2 Department of Neurology, Affiliated Zhongda Hospital of Southeast University, and the Institute of Neuropsychiatry of Southeast University, 87 Dingjiaqiao Road, Nanjing, Jiangsu, 210009, China

**Keywords:** Amnestic mild cognitive impairment, Functional magnetic resonance imaging, Amplitude of low-frequency fluctuation, Interleukin-1 beta, Cognition

## Abstract

**Purpose:**

The inflammatory response has been associated with the pathogenesis of Alzheimer’s disease (AD). The purpose of this study is to determine whether the rs1143627 polymorphism of the interleukin-1 beta (IL-1β) gene moderates functional magnetic resonance imaging (fMRI)-measured brain regional activity in amnestic mild cognitive impairment (aMCI).

**Methods:**

Eighty older participants (47 with aMCI and 33 healthy controls) were recruited for this study. All of the participants were genotyped for variant rs1143627 in the *IL1B* gene and were scanned using resting-state fMRI. Brain activity was assessed by amplitude of low-frequency fluctuation (ALFF).

**Results:**

aMCI patients had abnormal ALFF in many brain regions, including decreases in the inferior frontal gyrus, the superior temporal lobe and the middle temporal lobe, and increases in the occipital cortex (calcarine), parietal cortex (Pcu) and cerebellar cortex. The regions associated with an interaction of group X genotypes of rs1143627 C/T were the parietal cortex (left Pcu), frontal cortex (left superior, middle, and medial gyrus, right anterior cingulum), occipital cortex (left middle lobe, left cuneus) and the bilateral posterior lobes of the cerebellum. Regarding the behavioral significance, there were significant correlations between ALFF in different regions of the brain and with the cognitive scores of each genotype group.

**Conclusions:**

The present study provided evidence that aMCI patients had abnormal ALFF in many brain regions. Specifically, the rs1143627 C/T polymorphism of the *IL1B* gene may modulate regional spontaneous brain activity in aMCI patients.

## Background

Alzheimer’s disease (AD) is the most common form of dementia worldwide. It is characterized by two microscopic neuropathologic hallmarks - senile plaques composed of amyloid beta (Aβ) and neurofibrillary tangles composed of hyperphosphorylated tau. Mild cognitive impairment (MCI) is considered as an intermediate clinical state between normal aging and dementia [1]. According to the impaired domains of cognitive function, MCI can be divided into amnestic mild cognitive impairment (aMCI) and nonamnestic MCI [1]. aMCI is commonly regarded as the prodromal phase of AD, with 10 times the annual conversion rate of normal aging [2].

As research of reliable biomarkers of AD advances, brain Aβ-plaque deposition and neurodegeneration have been labeled as the biomarker categories in AD [3]. However, the use of a more extensive biomarker profile, rather than any single measure, may help improve the sensitivity and selectivity of predicting AD earlier [4]. In addition to senile plaques and neurofibrillary tangles, the Neuroinflammation Working Group has reached a consensus that inflammation is involved in the pathogenesis of AD [5]. Biomarkers, such as cytokines, have already been assessed [6,7]. The proinflammatory cytokine interleukin-1 beta (IL-1β) is a mental component in the inflammatory pathway and is overexpressed in the brain of AD patients. The gene for *IL1B* maps to chromosome 2q14. Several polymorphisms of this gene have been recognized [8]. Among these polymorphisms, the −31 T > C (rs1143627) polymorphism in the promoter region affects *IL1B* gene expression particularly.

Functional neuroimaging techniques, which have been utilized in the study of AD/MCI, mainly include single-photon emission computed tomography (SPECT), positron emission tomography (PET) and blood oxygenation level-dependent (BOLD) functional magnetic resonance imaging (fMRI). Resting-state fMRI has been developed as a new branch of this field. Compared with SPECT/PET, resting-state fMRI has an advantage in that there is no radiation exposure. Biswal and colleagues have demonstrated that spontaneous low-frequency (typically 0.01 to 0.08 Hz) fluctuations of the human brain, measured by resting state fMRI, are physiologically meaningful [9]. An approach to measure the amplitude of low-frequency fluctuation (ALFF) of BOLD signals in early AD has been developed to explore regional neural function by Zang and colleagues [10]. To date, ALFF has been widely applied to the study of different brain disorders, including epilepsy [11], schizophrenia [12,13], drug addiction [14], posttraumatic stress disorder (PTSD) [15], attention deficit hyperactivity disorder (ADHD) [16,17], multiple sclerosis (MS) [18], and aMCI [19]. This study aimed to determine whether rs1143627 moderates fMRI-measured brain regional activity in aMCI.

## Methods

### Study participants

A total of 47 aMCI patients and 33 healthy controls were recruited from the Affiliated Zhongda Hospital of Southeast University and communities in Nanjing, China. All of the participants, who were Han Chinese and right-handed, were interviewed by two trained senior neurologists. This study was approved by the Southeast University Ethics Committee and informed consent was obtained from all of the participants.

A diagnosis of aMCI, including single domain (the impairment involves only the memory domain) and multiple domain (impairment in the memory domain plus impairments in at least one other cognitive domain), was made following the recommendations of Petersen [20] and others [21], including (1) a subjective memory complaint, preferably corroborated by an informant, (2) an objective memory impairment, such as a score of less than or equal to 1.5 SD of age-adjusted and education-adjusted norms on the 20-min delayed recall of auditory verbal learning test (AVLT) (the cutoff was ≤4 correct responses on 12 items for ≥8 years of education), (3) normal general cognitive functioning, measured by a mini-mental state examination (MMSE) score of 24 or higher, (4) a clinical dementia rating scale (CDR) of 0.5, with at least a 0.5 in the memory domain, (5) normal or minimal impairment in the activities of daily living (ADL), a score of 20 to 26, and (6) absence of dementia, or not sufficient to meet the National Institute of Neurological and Communicative Disorders and Stroke and the Alzheimer's Disease and Related Disorders Association (NINCDS-ADRDA) criteria for AD. Participants were excluded if they had a past history of known stroke (modified Hachinski score >4), alcoholism, head trauma, Parkinson’s disease, epilepsy, major depression (excluded by the self-rating depression scale) or any other neurological or psychiatric illness (excluded by clinical assessment and case history), major medical illness (for example, cancer, anemia, thyroid dysfunction), or severe visual or hearing loss.

Control participants were required to have a CDR of 0, a MMSE score ≥26, and a delayed recall score >4 for those with 8 or more years of education.

### DNA isolation and SNP genotyping

Four milliliters of peripheral blood from each participant was collected into EDTA-containing vacutainer tubes and stored at −80°C. Genomic DNA was isolated using the Wizard Genomic DNA purification Kit (Promega, Madison, WI, USA) according to the manufacturer’s protocol. The PCR primers were designed using Genotyping Tools (Sequenom, Hamburg, Germany) and MassARRAY Assay Design software (Sequenom) and were synthesized by Invitrogen China (Beijing, China). PCR amplification reactions (5 μl) were performed in standard 384-well plates using 10 ng genomic DNA, 0.5 U of Taq polymerase (HotStarTaq, Qiagen, Valencia, CA, USA), 0.1 μl of 25 mM each dNTP and 0.5 pmol of each PCR primer. PCR thermal cycling was carried out for 4 min at 94°C, followed by 45 cycles of 20 s at 94°C, 30 s at 56°C, 1 min at 72°C and then 3 min at 72°C. Next, 0.5 U of shrimp alkaline phosphatase (SAP; Sequenom) and 0.17 μl of buffer were added to the PCR reaction products and were incubated for 20 min at 37°C, then inactivated for 5 min at 85°C to get rid of free dNTP. Post-PCR reactions, also called single base extension (SBE) reactions, which were performed in a final volume of 9 μl containing 0.804 μl of each Primer Mix, 0.041 μl of iPLEX enzyme, and 0.2 μl of Terminator, and consisted of 40 cycles of denaturation at 94°C and a final 3 min extension step at 72°C. The products of the iPLEX reaction were purified by 6 mg Clean Resin (Sequenom) and were then spotted on a SpectroChip (Sequenom). Data were processed and analyzed by MassARRAY TYPER 4.0 software (Sequenom).

### MRI scanning

All of the participants were scanned with a General Electric 1.5 Tesla scanner (General Electric Medical Systems, Miwaukee, WI, USA) by two experienced doctors from the radiology department following a standard imaging protocol. The participants lay supine with their heads snugly fixed by a belt and pads were used to minimize head motion. Functional images (T2* weighted images) were obtained using a GRE-EPI pulse sequence with the following parameters: 30 contiguous axial slices, slice thickness/gap = 4.0/0 mm, in-plane resolution = 3.75 × 3.75 mm^2^, TR = 3000 ms, TE = 40 ms, flip angle = 90°, acquisition matrix = 64 × 64, FOV = 240 × 240 mm. This acquisition sequence generated 142 volumes in 7 min and 6 s. In addition, three-dimensional T1-weighted axial images covering the whole brain were obtained using a spoiled gradient echo (SPGR) sequence (TR = 9.9 ms, TE = 2.1 ms, slice thickness = 2.0 mm, gap = 0 mm, flip angle = 15°, FOV = 240 × 240 mm, acquisition matrix = 256 × 192). Participants were instructed to keep their eyes closed, bodies aplanatic and not to think systematically or fall asleep during the scanning.

### MRI data preprocessing

The first eight volumes of each functional time course were discarded to allow for T1 equilibrium and to allow the participants to adapt to the scanning conditions. Slice timing, head motion correction, and spatial normalization were conducted using SPM5 (http://www.fil.ion.ucl.ac.uk/spm). Participants with head motion of more than a 3.0 mm maximum displacement in any direction (x, y, and z) or 3.0° of angular motion throughout the course of the scan were excluded from this study. The resulting images were spatially smoothed (full width at half maximum (FWHM) = 8 mm) using SPM 5. Then, REST software (http://resting-fmri.sourceforge.net) was used for linear trend removal temporal band-pass filtering (0.01 ~ 0.08 Hz) [9,22].

### ALFF calculation

ALFF was calculated using REST software similar to that used in previous studies [17,23,24]. Briefly, for a given voxel, after image preprocessing, the time series of resultant images was first converted to the frequency domain using a Fast Fourier Transform and the power spectrum was then acquired. The square root was computed at each frequency of the power spectrum and averaged between 0.01 and 0.08 Hz, and this averaged square root was termed ALFF. For standardization, a whole-brain mask was created by removing the background and other tissues outside the brain in the anatomical images using MRIcro software (http://www.mricro.com) [25-27].

### Voxelwise-based gray matter volume correction

To control for possible differences in ALFF that may be explained by differences in gray matter distribution between participants and to isolate the functional changes in components that cannot be attributed to anatomical differences, and thus are likely due to genuine functional differences, our study adopted estimates of a voxel’s likelihood of containing gray matter as a covariate (nuisance variable) in the analysis of the resting-state functional data [28]. First, voxel-based morphometry (VBM) [29,30] was used to explore gray matter volume maps of each participant. Second, the maps were transformed into the same standard space as the resting-state fMRI images using affine linear registration [31]. Finally, these resulting voxelwise gray matter volume maps were input as covariates in the analysis of the functional data. Voxelwise-based gray matter volume correction was used for each participant. The ALFF corrected by the voxelwise-based gray matter volume was then analyzed as follows.

### Statistical analysis

To determine the effects of group and genotype on ALFF, we performed a two-way analysis of variance (ANOVA) on a voxel-by-voxel basis with groups (aMCI patients and healthy controls) and genotypes (CC, CT and TT). To further explore the details of those clusters showing significant main effects and interactions, post hoc *t* tests were performed. All the statistical maps were corrected for multiple comparisons using a significance level of *P* value <0.05, based on Monte Carlo simulations (parameters: single voxel *P* value = 0.05, a minimum cluster size of 10503 mm^3^, FWHM = 8 mm, with mask. See program AlphaSim by D. Ward, and http://afni.nimh.nih.gov/pub/dist/doc/manual/AlphaSim.pdf). Finally, we performed a correlative analysis between the neuropsychological test scores and the ALFF values of the clusters showing significant interactions between group and genotype (*P* <0.05).

## Results

### Neuropsychological data

The demographic and neuropsychological data for all the participants are shown in Table 1. There were no significant differences in gender, age, education levels between the two groups. Compared with the controls, the aMCI participants showed deficits in memory (auditory verbal learning test delayed recall and Rey-Osterrieth complex figure test delayed recall), construction (clock drawing test), attention/psychomotor speed (trail making test A, symbol digit modalities and digit span test), and executive functions (trail making test B). Further comparisons of the neuropsychological battery between the three genotypes of aMCI patients showed no significant differences, all *P* >0.05 (Table 2).

**Table 1:** Demographic and neuropsychological data between aMCI group and healthy control group

**Items**	**aMCI group(n = 47)**	**Control group(n = 33)**	***P*value**
Gender (male: female)	28:19	18:15	0.654○
Age (years)	71.957 ± 4.777	72.848 ± 3.392	0.662△
Education levels (years)	15.894 ± 11.429	14.742 ± 2.889	0.124△
Clinical dementia rating (CDR)	0.5	0	—
Mini mental state exam (MMSE)	26.979 ± 1.525	28.182 ± 1.334	0.001*△
Auditory verbal learning test delayed recall	2.894 ± 1.747	7.970 ± 1.845	0.000*△
Rey-Osterrieth complex figure test delayed recall	10.787 ± 7.395	16.578 ± 7.009	0.002*△
Trail making test A (seconds)	94.404 ± 34.518	73.818 ± 29.591	0.006*△
Trail making test B (seconds)	189.447 ± 77.565	139.849 ± 43.953	0.003*△
Symbol digit modalities test	26.575 ± 9.930	34.000 ± 10.161	0.003*△
Clock drawing test	8.192 ± 1.676	9.000 ± 1.047	0.011*△
Digit span test	12.106 ± 2.119	13.091 ± 2.127	0.027*△

Note: data are presented as mean ± SD. *indicates had statistical difference between groups, *P* value <0.05. ○: The *P* value was obtained by Pearson chi-square test. △: The *P* value was obtained by Mann–Whitney *U* test, which was used here due to the data not being normally distributed.

**Table 2:** N**europsychological data between three genotype groups in aMCI patients**

**Items**	**CC(n = 13)**	**CT(n = 23)**	**TT(n = 11)**	***Chi-square***	***P***
		**mean rank**			
Mini-mental state exam (MMSE)	22.62	27.48	18.36	3.714	.156
Auditory verbal learning test delayed recall	26.00	21.33	27.23	1.863	.394
Rey-Osterrieth complex figure test delayed recall	26.58	21.74	25.68	1.254	.534
Trail making test A (seconds)	23.46	21.78	29.27	2.250	.325
Trail making test B (seconds)	20.15	24.85	26.77	1.561	.458
Symbol digit modalities test	24.00	25.48	20.91	.829	.661
Clock drawing test	20.50	26.43	23.05	1.747	.418
Digit span test	27.88	25.11	17.09	4.103	.129

Note: The P value was obtained by Kruskal-Wallis test (a type of nonparametric test) as the statistical test, as the data of the three groups were not normally distributed.

### The influence of diseases × genotypes on ALFF

**Figure 1:**
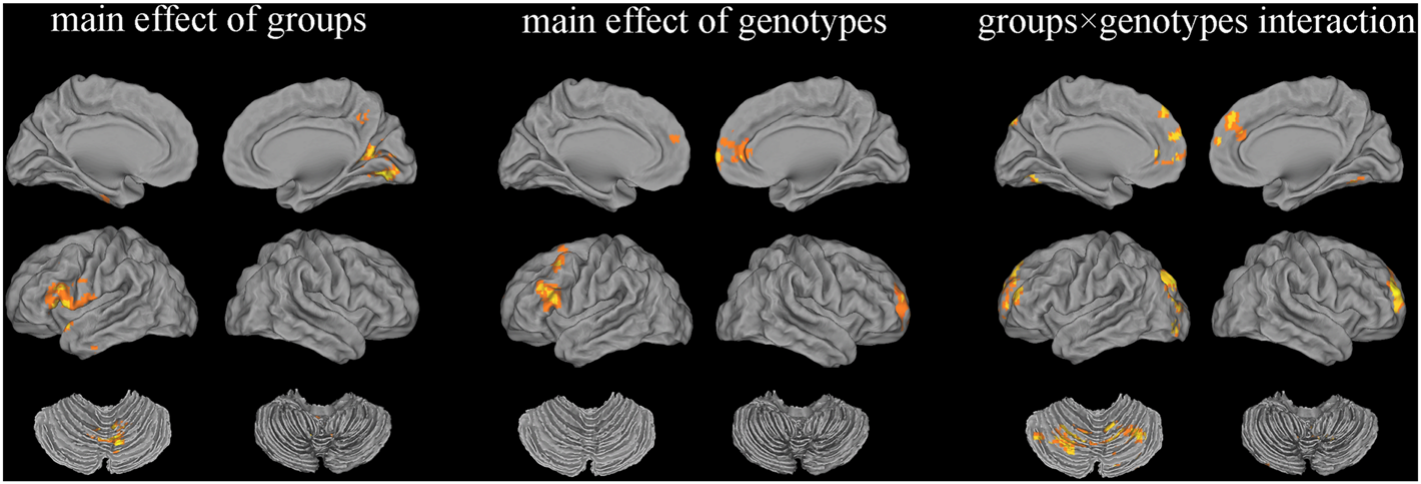
**Groups × genotypes ANOVA of ALFF. Thresholds were set at a corrected*P*<0.05, determined by Monte Carlo stimulation.**

(1) Brain regions showing a significant main effect of groups were identified in the frontal cortex (left inferior gyrus), temporal cortex (left superior and middle lobes), parietal cortex ((right precuneus (Pcu)), occipital cortex (right calcarine) and cerebellar cortex (right posterior lobe), while main effects of genotypes were found only in the frontal cortex (bilateral middle, left inferior and right superior gyri, right anterior cingulum). In particular, regions associated with an interaction of groups × genotypes were found in the parietal cortex (left Pcu), frontal cortex (left superior, middle, and medial gyri, right anterior cingulum), left middle occipital lobe and the bilateral posterior lobes of the cerebellum (for details see Table 3 and Figure 1).

**Table 3:** Groups × genotypes ANOVA of ALFF

**Brain region**	**BA**	**Peak MNI coordinates (mm)**			**Peak F value**	**Cluster size**
		**x**	**y**	**z**		
**(1) Main effect of groups**						
L inferior frontal gyrus/superior temporal lobe/middle temporal lobe	22/38/45	−57	21	21	26.78	23841
R calcarine/precuneus/cerebellum posterior lobe	7/18	6	−63	−18	12.27	15120
**(2) Main effect of genotypes**						
L middle frontal gyrus/inferior frontal gyrus	9/45	−57	21	18	19.53	13851
R medial frontal gyrus/superior frontal gyrus/ anterior cingulum	10	6	63	6	12.88	13608
**(3) Groups × genotypes interaction**						
L superior frontal gyrus/middle frontal gyrus/ medial frontal gyrus/R anterior cingulum	9/32/46	−54	27	21	13.14	19278
L middle occipital lobe/cuneus/precuneus	19	−21	−96	24	9.34	10557
B cerebellum posterior lobe	—	−24	−60	−24	8.56	18468

Note: a corrected threshold by Monte Carlo stimulation at *P* <0.05. Cluster size is in mm3. BA, Brodmann's area; B, bilateral; L, left; MNI. Montreal Neurological Institute; R, right.

### The influence of factors on changes in ALFF

**Figure 2:**
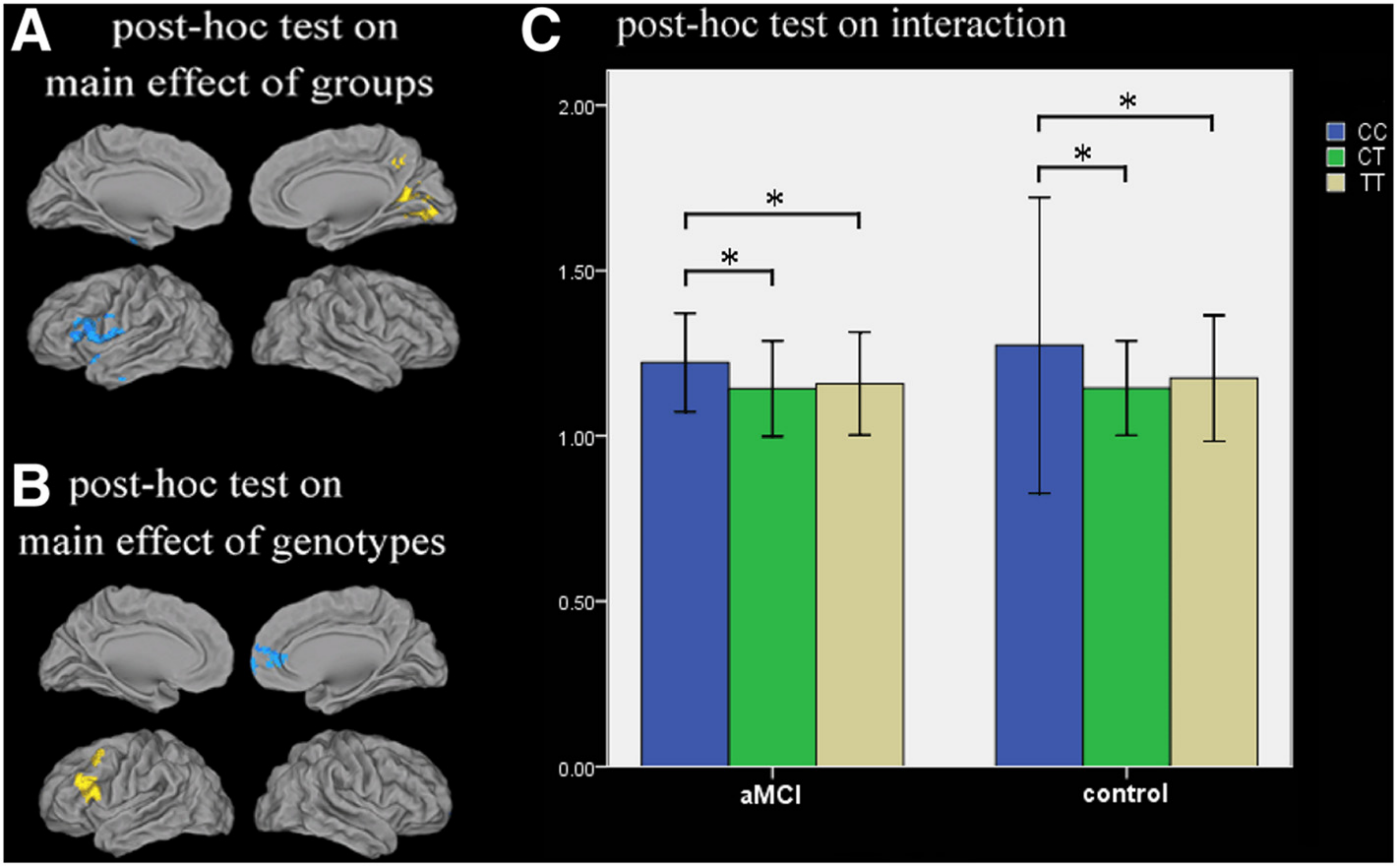
**Post hoc test.** Thresholds were set at a corrected *P* <0.05, determined by Monte Carlo stimulation. (**A**) Compared with controls group, aMCI group showed increased ALFF in right Pcu/calcarine, while decreased ALFF in left inferior frontal gyrus and superior/middle temporal lobe (yellow: aMCI >control, blue: aMCI <control). (**B**) Compared with CT genotype, CC genotype showed increased ALFF in left middle/inferior frontal gyrus, and compared with TT genotype, CC genotype also showed increased ALFF in right medial/superior frontal gyrus and right anterior cingulum, while there were no significance between CT and TT genotype (yellow: CC >CT, blue: CC >TT). (**C**) Further analysis of interaction between groups and genotypes found that both in aMCI and control groups, CC genotype showed significant higher ALFF than CT/TT genotype. The ALFF value for each genotype in controls group was higher than that in aMCI group based on visual comparisons (no significant difference in statistics). *P <0.05.

Post hoc test: a) Compared to the control group, the aMCI group showed decreased ALFF in regions of the dominant hemisphere, including in the left inferior frontal gyrus and the left superior and middle temporal lobe, while increased ALFF was shown in the right Pcu and calcarine regions (Figure 2A). b) Compared to the CT genotype, the CC genotype showed increased ALFF in the left middle and inferior frontal gyri, and compared to the TT genotype, the CC genotype also showed increased ALFF in right medial and superior frontal gyri and the right anterior cingulum, while there were no significant differences between the CT and TT genotypes (Figure 2B). c) Further analysis of the interactions between groups and genotypes revealed that in both the aMCI and control groups, the CC genotype showed significantly higher ALFF than the CT and TT genotypes. In addition, we found that the ALFF value for each genotype in the control group was higher than that of the aMCI group, based on visual comparisons (no significant difference in statistics) (Figure 2C).

### Correlations between ALFF values and behavioral scores

**Figure 3:**
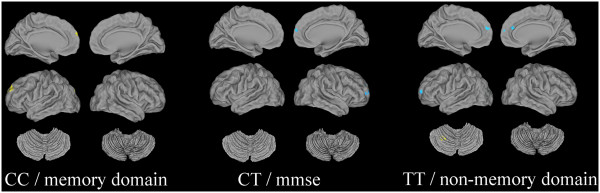
**Correlations between ALFF and neuropsychological tests.** Thresholds were set at a corrected *P* <0.05, determined by Monte Carlo stimulation (yellow: positive correlation; blue: negative correlation).

Correlations between the ALFF values of the clusters showing significant interactions with the neuropsychological tests scores are shown in Figure 3. There was a positive correlation in the left frontal cortex between ALFF and behavioral tests in the memory domain in the CC genotype, and a negative correlation in the right frontal cortex between ALFF and MMSE in the CT genotype, while in the TT genotype, we found a negative correlation in the bilateral frontal cortex and a positive correlation in the left posterior cerebellum between ALFF and the nonmemory domains.

## Discussion

In this imaging genetics analysis, we investigated for the first time the effect of rs1143627 (−31 T > C) loci polymorphism of *IL1B* gene on brain regional spontaneous activity in aged subjects presenting with aMCI and normally aging participants. We found that, among the CC, CT, and TT genotypes, many brain regions showed significant differences in ALFF between the aMCI patients and the controls. Interestingly, we found that the regions of interaction between the genotypes and the groups showed some overlap with the default mode networks, including the Pcu, the superior frontal gyrus, the anterior cingulum cortex (ACC) and some other frontal regions. Further behavioral significance suggested a role for the rs1143627 polymorphism in regional neuronal activity that has some effects on cognitive aging.

The protein IL-1β, encoded by a 7.5 kb gene with seven exons, is regulated both by distal and proximal promoter elements [32]. The promoter SNP locus rs1143627 (−31 T > C) is located in a TATA-box motif, which markedly affects DNA-protein interactions *in vitro*[33], and the change from −31 T to -31C may disrupt the TATA-box, leading to potential reduced or abolished transcriptional activity of the promoter [34,35].A genome-wide association study (GWAS) confirmed that −31 T > C had the highest LOD score, providing strong, unbiased evidence that this SNP is functional and that IL-1β mRNA expression is a heritable trait [36].

It has been demonstrated both *in vivo* and *in vitro* that basal IL-1β expression is involved in the physiological long-term potentiation (LTP), a process believed to underlie certain forms of learning and memory [37]. At sufficient concentrations, IL-1β has an inhibitory effect on LTP in many regions of the hippocampus [38]. Additionally, the injection of lipopolysaccharide (LPS), a potent inducer of IL-1β expression, into the CA1 regions of the rat hippocampus results in learning and memory deficits [39]. Postmortem brain tissue from patients with early-stage AD showed significantly increased expression of caspase-1 (CASP1), also known as IL-1β converting enzyme (ICE) [40]. A previous meta-analysis of cytokines found a significantly higher concentration of IL-1β in the peripheral blood of AD patients [6]. A case–control study on CSF concluded that intrathecal inflammation precedes the development of AD [41], and found the levels of IL-1β were significantly correlated with the levels of tau and Aβ in patients with MCI who had progressed to AD at the follow-up nine months later. Furthermore, it was found the IL-1β levels were correlated to MMSE at both baseline and follow-up assessments. There have been many associating *IL1B* gene SNPs with the risk of occurrence of AD, but the results are controversial (see http://www.alzgene.org/). A previous genetic association study of Chinese participants did not detect any genotypic or allelic frequency differences in *IL1B* -31C/T between an AD group and a control group, and concluded that the -31C/T polymorphism was not a risk factor for AD [42], while the result needs to be further confirmed.

Our study showed decreased ALFF in the inferior frontal gyrus, the superior temporal lobe and the middle temporal lobe in aMCI patients, which was consistent with previous hypoperfusion shown by SPECT and hypometabolism shown by PET in aMCI/AD [43,44]. It is worth noting that the frontal and temporal lobes, together with the parietal and occipital cortices, were previously identified with more accumulation of ^11^C-Pittsburgh compound B (PIB), consistent with brain regions containing Aβ deposits [45]. A previous resting state fMRI study also found that reductions in regional activation [46,47], regional homogeneity [10,48] and functional connectivity [49-52] were associated with both AD and MCI patients. A recent combined structural and resting-state fMRI study [53] found broad frontotemporal grey matter loss in MCI patients by voxel-based morphometry (VBM) analysis, and found regions of decreased connectivity to the posterior cingulated cortex (PCC), which provides further support for our findings. In the current study, we also observed increased ALFF in the aMCI group, including in the occipital cortex (calcarine), parietal cortex (Pcu) and cerebellar cortex. Jia and colleagues [19] found increased ALFF/fractional ALFF (fALFF) activity in aMCI patients in several occipital regions, which is consistent with the present study. A previous PET study [54] showed a significant increase in metabolism in the occipital cortex in aMCI brains. Researchers have also observed that baseline aMCI patients have increased cerebellar ALFF compared to controls [55]. Thus, our results of ALFF in aMCI suggested that aMCI patients might have relatively conserved spontaneous brain activity in these regions. The increased ALFF in the Pcu of aMCI patients in this study was in contrast to previous studies, which showed a decrease in ALFF. A structural MRI study showed that Pcu undergoes significant atrophy in MCI and mild AD [56]. In addition, lower functional connectivity within the default mode network (DMN) in the Pcu and PCC was detected in AD patients by an independent component analysis (ICA) [57]. The PCC and Pcu of aMCI patients displayed decreased ALFF/fALFF in a recent study [19]. We found similar results regarding Pcu in the present study. A recent resting state fMRI study [58] identified PCC and Pcu hyperfunctional connectivity at baseline in aMCI subjects, and a substantial decrease in these connections was evident at follow-up, compared to matched controls. Additionally, Jones *et al*. [59] analyzed task-free fMRI data with both ICA and seed-based analysis, identifying the posterior DMN as having a decline in connectivity, while an increase in connectivity was observed in the Pcu when atrophy correction was applied. A previous study [60] demonstrated that endogenous neuronal activity regulated the regional concentration of interstitial fluid Aβ levels, which drive local Aβ aggregation. It is uncertain whether some regions with early increased regional neural activity and hyperfunctional connectivity could be a mechanism of compensation for AD pathology or represent a more proximate event leading to cognitive dysfunction. In summary, our results suggested that aMCI patients had abnormal ALFF in their intrinsic brain activity.

In this study, we observed that the rs1143627 C/T genotype had main effects on the frontal cortex, including the superior, middle, inferior and medial frontal gyri, and the ACC. SNPs in the *IL1B* gene were shown to have effects on the responsiveness of the amygdala and ACC to emotional stimulation in major depression [61]. Additional brain regions that had interactions with groups were in the frontal, parietal and occipital cortices and in the posterior lobe of the cerebellum. A recent genetic neuroimaging study [62] detected the effect of the *IL1B* gene on frontal cortex function in schizophrenia. A mammalian genome study identified *IL1B* gene polymorphisms that modulated scrapie (a type of neurodegenerative disease) susceptibility in sheep and goats, and found IL-1β expression in the cerebellum [63]. These studies supported our findings of the genetic effects of rs1143627 on different brain regions. In addition, the CC genotype showed significantly higher ALFF values than the CT and TT genotypes in both aMCI and control participants, and each genotype showed different or completely opposite results in the association between ALFF and cognitive scores. This study did not find the frequency of the three genotypes between groups, consistent with a previously mentioned study [42]. We did not find any significant effects of the genotypes on cognition in aMCI patients. This was not in contradiction to the behavioral significance, as the pathophysiological process of AD is thought to begin many years, even decades, before the onset of clinical dementia [64,65]. Thus, further large-scale association replicated and longitudinal studies are needed.

There were biological and technical limitations to this study that must be acknowledged. First, there was biological and clinical heterogeneity in the sample of aMCI participants as recruitment was based only on clinical criteria. Some participants may not display AD pathology, contaminating the sample with non-AD cases. The heterogeneity could be minimized through a combination of CSF and PET biomarkers. Second, as a result of the limitation of the sample size, further adjustment for multiple testing outside the domain of imaging will be necessary and replication in independent samples is also required to further establish the gene-imaging phenotype association. Third, previous resting state fMRI studies [66,67] have demonstrated very moderate test-retest reliability based on Monte Carlo simulations and, because calculation methods will be improved with time, new analysis methods could be applied to future studies.

## Conclusions

In this study, we provide evidence that aMCI patients had abnormal ALFF in many brain regions, including in the Pcu, the calcarine, the inferior frontal gyrus and the superior and middle temporal lobe. In particular, we showed that the rs1143627 C/T polymorphism might have an effect on spontaneous regional brain activity in aMCI patients.

## Abbreviations

ACC: Anterior cingulum cortex; AD: Alzheimer’s disease; ADHD: Attention deficit hyperactivity disorder; ADL: Activities of daily living; ALFF: Amplitude of low-frequency fluctuation; aMCI: amnestic mild cognitive impairment; Aβ: amyloid beta; AVLT: Auditory verbal learning test; BOLD: Blood oxygenation level-dependent; CASP1: Caspase-1; CDR: Clinical dementia rating scale; DNM: Default mode network; fALFF: fractional ALFF; fMRI: Functional magnetic resonance imaging; FWHM: Full width at half maximum; GWAS: Genome-wide association study; ICA: Independent component analysis; ICE: IL-1β converting enzyme; IL-1β: Interleukin-1 beta; LPS: Lipopolysaccharide; LTP: Long-term potentiation; MCI: Mild cognitive impairment; MMSE: Mini-mental state examination; MS: Multiple sclerosis; NINCDS-ADRDA: National Institute of Neurological and Communicative Disorders and Stroke and the Alzheimer's Disease and Related Disorders Association; PCC: Posterior cingulated cortex; Pcu: Precuneus; PET: Positron emission tomography; PIB: Pittsburgh compound B; PTSD: Posttraumatic stress disorder; SNP: Single nucleotide polymorphism; SPECT: Single-photon emission computed tomography; SPGR: Spoiled gradient echo; VBM: Voxel-based morphometry.

## Competing interests

The authors declare that they have no competing interests.

## Authors' information

Zhijun Zhang is a professor of Southeast University, the person in charge of the Department of Neurology, affiliated ZhongDa Hospital of Southeast University and the Institute of Neuropsychiatry of Southeast University.

She has been doing research on amnestic mild cognitive impairment, both on gene and brain imaging studies, for more than seven years, and her papers have been published by journals such as *Biological Psychiatry*, the *Lancet* and so on.

## Authors’ contributions

Liying Zhuang analyzed the data, made figures and tables and wrote the manuscript. Xiaoyan Liu, Xiaohui Xu, and Hao Shu helped analyze the data. Chunxian Yue designed the study. Feng Bai helped analyze the data and revise the manuscript. Hui Yu and Yongmei Shi contributed to the study conception and design and acquisition of data. Zhijun Zhang contributed to the study conception and design and manuscript revision. All authors provided editorial assistance and have read and approved the final version of the manuscript.
